# Seasonal variation in harbour seal *(Phoca vitulina)* blubber cortisol - A novel indicator of physiological state?

**DOI:** 10.1038/srep21889

**Published:** 2016-02-24

**Authors:** Joanna L. Kershaw, Ailsa J. Hall

**Affiliations:** 1 Sea Mammal Research Unit, Scottish Oceans Institute, University of St Andrews, St Andrews, Fife. KY16 8LB, UK

## Abstract

Cortisol is one of the main glucocorticoid hormones involved in both the mammalian stress response, and in fat metabolism and energy regulation, making it of increasing interest as a biomarker for stress, health and overall physiological state. However, transient stress responses to animal handling and sampling may be important sources of measurement artefact when investigating circulating concentrations of this hormone in wildlife. Here, cortisol concentrations were measured in the plasma and, for the first time, in the blubber of live captured adult harbour seals (*Phoca vitulina*). Plasma cortisol concentrations were positively correlated with capture time, suggesting that they were largely driven by a stress response to the capture event. In contrast, blubber cortisol concentrations were shown not to be significantly affected by capture time and varied significantly by sex and by season, with higher concentrations during natural fasting periods of their life cycle, particularly during the moult. These results suggest that cortisol may play a key role in increased fat metabolism during highly energetically demanding periods, and that blubber concentrations have the potential to be used as physiological state indicators in phocid seals.

Wild animals encounter a diverse range of environmental stressors that can be described as any physical or psychological event that disrupts homeostasis^1^. Their responses to such stressors are largely hormonally mediated, particularly by the glucocorticoids^2^, primarily cortisol^2^. The adrenal secretion of cortisol in response to stressful psychological stimuli (e.g. predators) and physiological stimuli (e.g. nutrient limitation) is controlled by the hypothalamic-pituitary-adrenal (HPA) axis^3^, and can be either short-term (acute) or long-term (chronic).

In terrestrial mammals, as well as their role in the stress response, the glucocorticoids have also been proposed as long-term regulators of both energy intake and storage^4^. Glucocorticoids increase in circulation in response to energetic needs, and their levels are generally interpreted as indicators of allostatic load^5^. Cortisol is of particular interest in the regulation of whole body energy stores as it is involved in maintaining the balance between fat storage where triglycerides are deposited, and fat depletion where they are catabolised and released into circulation^6^,^7^. Overall, cortisol is known to increase lipolysis, stimulate gluconeogenesis, mobilise amino acids, and increase circulating concentrations of plasma proteins^8^,^9^. Cortisol concentrations could therefore also be used as indicators of overall physiological state and provide vital information on the health and resilience of a population as a whole. For these reasons, conservation biologists are increasingly using glucocorticoid hormone assessments to monitor stress and animal health to better inform management efforts^10^,^11^.

However, studying natural variation in glucocorticoid hormone concentrations in wild populations is inherently very difficult as the temporary capture and restraint necessary for sampling may alter the physiological parameters of interest. Animals are typically manually restrained or sedated for varying lengths of time, and it is well recognised that these sampling procedures are potentially highly stressful for the study animals^12^. It is therefore very important to appreciate the magnitude and duration of this stress response to the capture event as it may compromise the specific aims of different studies by masking any underlying variation in these hormones levels.

In pinniped research, animal handling cannot be avoided for the collection of physiological samples. Increases in circulating cortisol concentrations are characteristic of their stress response to such handling and physical restraint procedures^13^,^14^,^15^. Natural cycles due to reproductive and physiological condition^16^,^17^,^18^,^19^,^20^ may therefore be masked by the effect of handling on cortisol secretion. Sedation has been shown to reduce the stress response in phocids, such that hormone concentrations under these conditions are more likely to reflect basal values^14^,^15^. However, there is often a time delay between capture and sedation which is then reflected in the circulating cortisol concentrations post-sedation^15^.

Sampling other fluids, tissues and excreta instead of the plasma may therefore provide more representative baseline information on cortisol concentrations that could be less affected by the sampling procedure. For example, levels of glucocorticoid hormones, including cortisol, or their metabolites have been measured in various marine mammal matrices including the faeces^21^,^22^, saliva^23^, hair^24^,^25^ skin^26^, blow expirate^27^ and also in the blubber^28^,^29^. Here, we investigate the increases in plasma cortisol concentrations in wild caught harbour seals (*Phoca vitulina*) following capture and handling. We also investigate the presence and variation of cortisol in the blubber to assess its potential as an informative biomarker of physiological state after allowing for any capture stress effects. Determining the dynamics of cortisol throughout the life cycle of this species at its site of action, in the subcutaneous fat stores, would be a key step towards understanding the endocrine control of energy regulation in pinnipeds, and in establishing whether glucocorticoid concentrations in this matrix can be used as a potential marker to better understand health and condition in wild populations.

## Results

### The Effect of Capture Time

**Figure 1:**
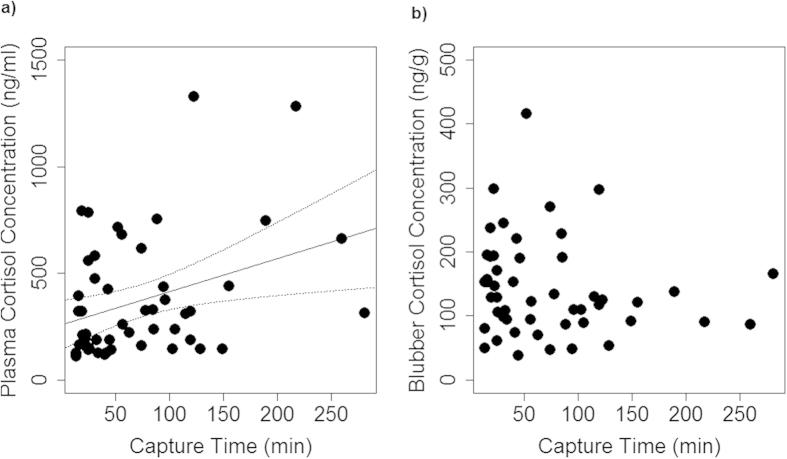
(**a)** Significant positive correlation between capture time and plasma cortisol concentrations over the 5 hour sampling period (linear regression model, adjusted R^2^ = 0.1, p = 0.01). (**b)** No significant correlation between blubber cortisol concentration and capture time over the same sampling period.

Plasma cortisol concentrations ranged between 102.10–1662.81 ng/ml. Capture times were recorded for 50 of the 85 individuals, and ranged from 14–281 min. Overall, there was a positive correlation between plasma cortisol and capture time (linear regression model, adjusted R^2^ = 0.1, p = 0.01), with large amounts of individual variation (Fig. 1a). In contrast, there was no correlation between blubber cortisol concentrations and capture time (linear regression model, adjusted R^2^ = −0.003, p = 0.37) (Fig. 1b).

### Variation in Blubber Cortisol

**Figure 2:**
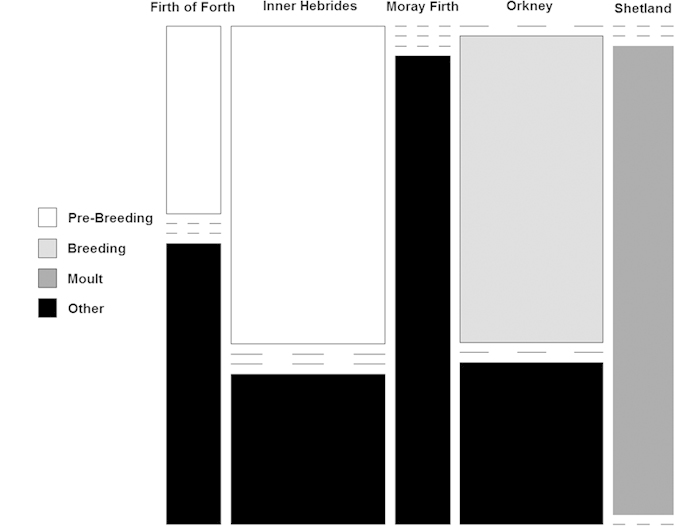
Plot showing the distribution of samples across the five sampling areas and over four seasons. Three of the areas (Firth of Forth, Inner Hebrides and Orkney) were sampled over two seasons while the other two areas (Moray Firth and Shetland) were sampled once.

Using the correction factor for the average blubber sample mass (0.2 g), the calculated limit of detection for the cortisol concentration in the blubber samples was approximately 4000 ng/g for this study, and measured concentrations ranged from 37.84 to 1553.58 ng/g. As only three of the five areas were sampled over two different seasons (Fig. 2), the respective effects of season and area were considered separately in the modelling process so as to take into account the potential variation as a result of season, and as a result of sampling area.

**Figure 3:**
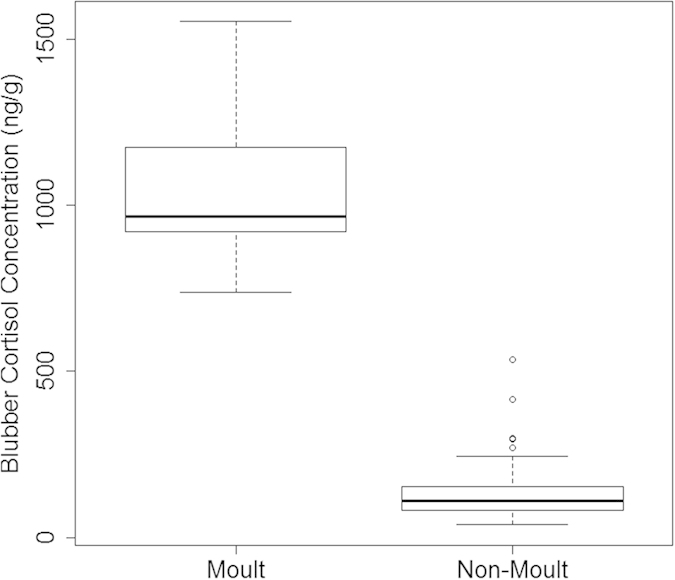
Blubber cortisol concentrations during the moult and over the rest of the year. Blubber cortisol concentrations were up to two orders of magnitude higher during the moult.

**Figure 4:**
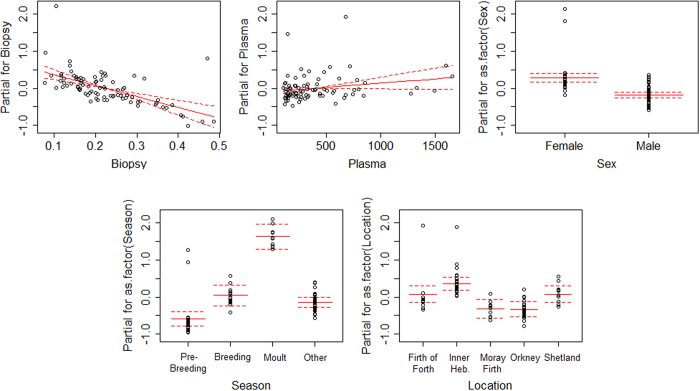
Blubber cortisol partial term plot for the final GLM (*glm(Blubber ~ Biopsy  *+* Plasma *+* as.factor(Sex) *+* as.factor(Season) *+* as.factor(Location), family *=* Gamma(link *=* log)*). Biopsy mass, sex, season and location were all statistically significant in the final model, but plasma cortisol concentration was not.

The best Generalised Linear Model following backwards variable selection for the blubber cortisol data revealed that biopsy mass, sex, season, and area were all statistically significant. Plasma cortisol concentration was retained in the final model, but was not statistically significant. Body condition and an interaction between sex and season were not included in the final model (Supplementary Table S1 online). Blubber cortisol concentrations were up to two orders of magnitude higher during the moult compared to the other seasons sampled (Kruskal-Wallis test, H = 28.4, p < 0.0001) (Fig. 3). The breeding season had the highest concentrations for both sexes excluding the moult, while concentrations were lowest immediately prior to the breeding season in May (p values < 0.003). Overall, males had significantly lower blubber cortisol concentrations than females (p < 0.0001). Finally, variation across the different areas revealed that the seals in the Inner Hebrides had significantly higher blubber cortisol concentrations than those in the Moray Firth and in Orkney (p values < 0.05) (Fig. 4).

Even when cortisol concentrations were corrected for extraction efficiency, biopsy mass had a significant effect on blubber cortisol in the final model (p < 0.001). In order to assess the potential bias of this effect on the model results, samples over 0.2 g were removed from the model as above this mass, it was seen from the extraction efficiency verifications that efficiency dropped from between 90–100% when sample masses increased over 0.2 g (Supplementary Fig. S3 online). When the samples over 0.2 g were removed from the analysis, 44 remained in the dataset from across all seasons and locations. The same modeling and variable selection processes were performed as before. Model selection revealed that the same covariates were included in this final model, with the same overall differences between the sexes, across seasons and between locations. Thus, using only using the results from the samples with high extraction efficiencies in the analysis did not affect the overall results.

## Discussion

The plasma cortisol concentrations showed considerable individual variation with higher concentrations recorded than in other studies on harbour seals published to date^17^,^19^,^30^,^31^,^32^, but within the ranges published for other phocid species^15^,^20^,^33^,^34^. The plasma concentrations measured were significantly positively correlated with capture time and therefore appear to represent different stages of a stress response to the capture and handling event. The same relationship between plasma cortisol concentrations and ‘elapsed time’ was observed in a population of bottlenose dolphins (*Tursiops truncatus*) in response to capture and handling^35^. Some of the individual variation in these results is likely to be due to confounding factors not controlled for in this study. The ELISA kit used had a 9% cross-reactivity with progesterone, and while there was no significant difference in the plasma concentrations between males and females, and the highest individual concentrations were measured in males in both matrices, it is possible that there is some interference with this hormone in the assay results. In addition, the reproductive histories for the adult females were unknown, and whilst none were sampled during lactation, differences in reproductive state may also be a potential confounder.

Assuming that the stress response is the same in harbour seals as other mammals, upon activation of the HPA pathway, adrenocorticotropin hormone (ACTH) is released from the anterior pituitary into the bloodstream which stimulates the adrenal cortex to produce glucocorticoids above baseline levels with a measurable increase in circulating levels within 3–5 minutes^36^. Hormone concentrations then continue to increase for 15–30 minutes after exposure to the stressor^36^, as appears to be the case in the seals sampled here. The termination of the pathway is controlled by the glucocorticoids themselves through a negative feedback loop such that stress-induced concentrations of glucocorticoids interact with their receptors in the hippocampus, hypothalamus and pituitary to suppress the continued activation of the pathway^37^. Circulating concentrations generally return to baseline levels within 60–90 minutes^36^. Under conditions where the stressor is chronic rather than acute however, these negative feedback signals are weak and the pathway remains activated for a longer period^38^.

Delayed negative feedback on the HPA axis was observed in northern elephant seals (*Mirounga angustirostris*) following the administration of exogenous ACTH^39^, and a similar delayed negative feedback mechanism may have taken place in the seals sampled here as there was no decrease in plasma concentrations in seals sampled after 90 minutes. However, a longitudinal sampling study design would be needed to confirm this. The large amount of individual variation across capture times could be due to individuals initially experiencing varying ‘stress loads’ at the time of sampling as a result of seasonal and sexual variation for example, which could then affect the extent to which they respond to the capture event. While only four animals were sampled over 2.5 hours (and showed considerable individual variation among their plasma cortisol concentrations), in general, the longer animals were held in nets, the higher their circulating cortisol concentration. Therefore, given the nature of typical capture methods at harbour seal haul outs that involve the retention of multiple individuals caught in a net at once, thus prolonging individual capture times, measuring baseline stress hormone levels in the plasma that are unaffected by these procedures is likely to be unattainable.

This study demonstrates that cortisol can be extracted from pinniped blubber in a similar way to cetacean blubber^28^,^29^, and quantified using a commercially available ELISA. The blubber cortisol concentrations reported here provide the first data on this hormone in marine mammal blubber biopsies from live animals, and demonstrate its potential variability in this matrix throughout the life cycle of a phocid seal. Here, full depth blubber biopsies were analysed, and further work to investigate differential deposition of this hormone through the blubber layer should be prioritised. In addition, tissue sample mass for extraction has to be chosen carefully for unbiased measurements of cortisol. Attempts were made to avoid the loss of tissue components that may affect the results (lipids, proteins and hormones) through excessive sub-sampling and handling of the biopsies by extracting them whole. However, the correlation between biopsy mass and cortisol concentration suggests that the extraction efficiency validations did not reflect the full extent to which extraction efficiency decreases with larger pieces of tissue. This is likely to be because with larger biopsies, tissue disruption is less effective and some hormone is not released from the matrix while the spiked cortisol added is already in solution and therefore readily available for extraction. In addition, the ratio of tissue mass to solvent volume which maximises extraction efficiency needs to be optimal, and the larger sample masses analysed here did not meet this ratio. Thus, it is recommended that even with the potential loss of other tissue components, biopsies should be sub-sampled for a sample mass of between 0.1 and 0.2 g to avoid underestimating the concentration of hormone in the tissue.

There was no significant relationship between capture time and cortisol concentration in the blubber over the 5 hour sampling period investigated here. This suggests that, within this time window, the blubber may be an appropriate tissue to sample in order to investigate cortisol concentrations in terms of natural physiological processes as it is much less sensitive to the acute stress response following a capture event in this species. Blubber cortisol concentrations were highest during the August moult. An increase in plasma cortisol has also previously been shown in moulting harbour seals^30^,^40^. It is thought that the moult is an energetically costly process for phocids^41^,^42^,^43^,^44^, as moulting animals need to supply nutrients, energy and oxygen to epidermal cells while maintaining a skin surface temperature that is optimal for hair growth^45^. In addition, they undergo periods of fasting as they haul out for longer to facilitate hair regeneration and to avoid thermal stress^45^ for the duration of the moult lasting between approximately 20 and 40 days^40^. For these reasons, harbour seals may have to draw on their energy reserves more during the moult than over the rest of the year, with the exception of lactation in females. Thus, increased circulating concentrations of cortisol, as well as increased concentrations in the blubber fat stores themselves over this time period could be required to facilitate the mobilisation of stored energy reserves.

Outside the moult, the highest blubber cortisol concentrations were measured during the breeding season in both males and females. Females had the highest concentrations overall. Lactating females greatly restrict their foraging in the early stages of lactation^46^, and therefore have to rely heavily on their fat reserves to meet the demands of lactation where increased lipid mobilisation is necessary for the production of fat-rich milk^47^. Thus, higher blubber cortisol concentrations leading up to pupping in females may aid in lipid mobilisation for milk production as well as energy release. Males also have a reduced food intake over the breeding season as they restrict their foraging range and spend more time making shorter and shallower dives accompanied by vocal displays thought to be associated with male mating behaviour^48^. Males spend time maintaining small territories and thus less time foraging^48^, so higher blubber cortisol concentrations leading up to breeding may be a result of increased demand on blubber energy stores over this period as well as the result of stressors associated with competing for territories and mating.

While foraging effort is reduced for both males and females during the breeding season, it appears that feeding does still take place^46^,^48^. This may explain why blubber cortisol levels are lower in the breeding season compared to the moult when animals remain hauled out for extended periods^49^, and foraging is reduced to a much greater extent such that reliance on the mobilisation of energy stores is more extreme. The lowest blubber cortisol concentrations were measured immediately prior to the breeding season in May, possibly as a result of individuals depositing fat stores instead of mobilising them in preparation for the short term fasting associated with the breeding season^46^,^48^.

There was significant variation in blubber cortisol concentrations between three of the five locations with significantly higher concentrations measured in seals captured in the Inner Hebrides compared to animals captured in the Moray Firth and in Orkney. Movement data from satellite relay dataloggers deployed on the animals from the Inner Hebrides (McConnell, B. pers. comm.) revealed that they made longer trips to distant foraging and haul out sites than seals from the other regions. This may indicate that short term fasting (3–5 days) during transit to and from these sites may also result in increased lipolysis and elevated blubber cortisol.

Adipose tissue has been shown to accumulate steroid hormones as they passively diffuse out of the blood through the capillaries that permeate the tissue^50^. As such, the cortisol concentrations measured in the blubber here may reflect circulating concentrations of this hormone through passive diffusion from the blood. The increase in blubber cortisol during the moult could be the result of increased perfusion of the tissue over this period which thus results in the accumulation of more hormone. However, recent data indicate that both the uptake of cortisol and its turnover in human adipose tissue is slow^51^. While phocid seal blubber is likely to be more highly vascularised than human adipose tissue, as cetacean blubber is more vascularised than typical adipose tissue^52^, and therefore perfused to a greater extent, the low lipophilicity of cortisol^53^ may mean that the uptake and turnover of this hormone in the blubber is still slow.

An alternative hypothesis is that the high blubber cortisol concentrations could be driven by cortisol production in the blubber itself, rather than solely as a result of passive diffusion of the steroids into the tissue from the blood. A body of literature highlights the importance of mammalian adipose tissue as an endocrine organ involved in both the production and metabolism of steroids (for a review see^54^ ). Cortisol is released from subcutaneous adipose tissue after its conversion from cortisone by the 11 β-hydroxysteroid dehydrogenase type 1 (11β-HSD1) enzyme in humans such that human adipose tissue contains glucocorticoids derived from both systemic circulation and local generation within the tissue itself^51^,^55^. Recent results in an ongoing project have demonstrated the presence of 11β-HSD1 mRNA transcripts in the blubber of phocid seals (Bennett, K. pers. comm.). Thus, if the same cortisone-cortisol shuttle in blubber tissue occurs in seals though the activity of this enzyme, this could be the primary source of the hormone during times of high energy demand to mobilise lipids. The blubber in phocids may therefore be acting as an endocrine organ by both producing and responding to hormone signals that are involved in regulating metabolism, and is not merely passively accumulating cortisol from the blood as has been suggested in cetaceans^28^,^29^. It is possible that cortisol concentrations in the blubber are derived from both circulating concentrations and local production of the hormone during periods of high energy demand. To further investigate this possibility, both the activity of the 11β-HSD1 enzyme, and the concentrations of cortisone should be measured in the tissue over the different life history stages of phocid seals.

To conclude, transient stress responses to capture and handling may be important sources of measurement artefact, and this work highlights the importance of measuring capture time as any variation in plasma cortisol levels as a result of life history stage would be affected by handling stress. This study has demonstrated that there appears to be considerable uptake and storage and/or metabolism of cortisol in blubber tissue, and as a result, it may play an important role in hormone homeostasis. The extreme changes in energy requirements throughout the life-cycle of harbour seals may explain the seasonal changes in blubber cortisol concentrations observed here. Cortisol concentrations were increased during periods of the year when the seals reduce their forging efforts and rely on stored blubber reserves for energy which suggests that cortisol may be important in lipolysis during fasting. Notwithstanding that blubber cortisol may be being produced locally, or whether blubber cortisol simply reflects circulating concentrations of this hormone through adrenal production, the concentration of cortisol in this matrix may be an important practical alternative for investigating longer term physiological changes of this hormone in phocids. Understanding the mechanisms by which glucocorticoids are stored and metabolised in the blubber of phocid seals is critical to further our understanding of the pleiotropic nature of blubber and its functional ecological role in shaping phocid seal life history strategy.

## Materials & Methods

### Sample Collection

Licensing: All research reported in this study was approved by the University of St Andrews Animal Welfare and Ethics Committee (AWEC) and was carried out under the Sea Mammal Research Unit’s Home Office licence number 70/7806 in accordance with the guidelines issued under the Animal (Scientific Procedures) Act 1986 and under personal licence number (PIL 80/1552) issued to Dr. Ailsa Hall. All research reported in this study was also licenced to be carried out in the field under the Marine (Scotland) Act 2010, Part 6 Conservation of Seals Research Licence issued to the Sea Mammal Research Unit.

Paired blubber biopsy and blood samples were collected simultaneously from 85 live captured adult harbour seals from five areas across Scotland between 2010 and 2014. Seals were sampled in Shetland, Orkney, the Moray Firth, the Firth of Forth and the Inner Hebrides between March and October, with repeated captures at different times of year in three of these five areas. The seals were captured in nets either on the haul-out sites or in the water. The ‘capture time’ was recorded for a subset of these animals (n = 50) as the time from when the animals were first disturbed off their haul out straight into capture nets, to the time at which they were sedated. This time therefore encompasses the entire duration of the stressor event from initial disturbance to sedation. Capture times varied based on the number of individuals caught in one capture attempt as each animal was immobilized and sampled in turn while other individuals caught at the same time were retained in hoop nets. The animals were sedated with Zoletil 100 (Virbac, France) at a dose rate of 0.5 ml/100 kg body weight intravenously. Immediately after the sedative was injected into the extradural vein, the syringe was replaced with a heparinized Becton Dickinson Vacutainer (Oxford, UK), and blood samples were collected. Blubber biopsies were taken from the flank area following local anaesthesia using subcutaneous injections of Lignol (Dechra, UK), between 5 and 10 minutes after the animal was sedated. A small incision was made through the skin, and a core biopsy punch was used to obtain a full depth blubber sample. The blubber samples were wrapped in aluminum foil, placed in individual plastic vials, and stored at −20 °C. The blood samples were centrifuged at 1,500*rcf* for 10 minutes and aliquots of the plasma collected and stored at −20 °C before analysis.

### Sample Analysis

#### Cortisol extraction from blubber biopsies

Given the similarities in the structure and physical properties of the steroid hormones, the same method has been used for the extraction of progesterone and cortisol from human adipose tissue^56^. For this reason, the extraction method developed by Kellar *et al.* (2006) for reproductive hormones from cetacean blubber samples was used here to extract cortisol from the blubber biopsies^57^. In order to avoid losing hormone through too much tissue handling and sub-sampling, all blubber biopsies were extracted whole. Masses ranged from 0.0768–0.4861 g (see Supplementary Information online).

#### Cortisol quantification in blubber biopsies and plasma samples

A commercially available Enzyme Linked Immunosorbent Assay (ELISA) (DRG International Inc. Cortisol ELISA EIA-1887) was the used for the quantification of cortisol in all samples. The concentrations were measured according to the ELISA kit instructions with a standard curve ranging between 0 and 800 ng/ml with a sensitivity of 2.5 ng/ml. The hormone concentrations in the samples were determined using a 4 parameter log-logistic model based on the standard curve. While none of the samples were at or below the limit of detection of the assay, any samples with a concentration higher than the highest standard (>800 ng/ml) were diluted with the 0 ng/ml standard and re-assayed to bring the concentration down onto the standard curve. All samples were assayed in duplicate and the mean hormone concentration reported. These means were reported as cortisol per wet weight of the biopsy for the blubber samples in ng/g, and as ng/ml for the plasma samples that were assayed unextracted^19^. While this ELISA has a 9% cross-reactivity with progesterone, as there was no significant difference between plasma cortisol concentrations measured in males and females (1 way ANOVA, DF = 1, F value = 1.73, p = 0.19), and as all 5 individuals with the highest plasma cortisol concentrations were males, and 7 of the top 10 highest plasma *and* blubber cortisol concentrations were also all males, we are not concerned that variation in progesterone concentrations have a large effect on these data.

High, medium and low concentration samples were used to calculate intra-assay and inter-assay coefficients of variation^58^,^59^. Percentage coefficients of variation (% CV) of <20% and <10% were set as the acceptable limits for the inter- and intra-assay % CV respectively^60^. The inter-assay coefficients of variation ranged between 5.9% and 17.0% for the blubber extracts, and between 4.3% and 13.9% for the plasma samples. The mean intra-assay coefficients of variation for the blubber extracts and plasma samples were 1.4% and 0.6% respectively.

### Verifications

#### Parallelism Assays

Five plasma samples and six blubber extracts were serially diluted from undiluted to 1/2, 1/4, and 1/8 with the 0 ng/ml cortisol standard provided in the ELISA kit. Parallelism between these dilutions and the standard curve was assessed and provided strong evidence of the reliable determination of hormone concentrations in these samples (see Supplementary Fig. S1 online).

### Matrix Effect Tests

As the ELISA kit used here was designed for use with serum or plasma samples, the compatibility of the kit with extracts resuspended in PBS with 1% bovine γ globulin was assessed (see Supplementary Fig. S2 online). The results showed that matrix effects are minimal and this sample diluent is therefore compatible with the immunoassay.

#### Cortisol Recovery from Spiked Samples

As the blubber biopsies were of varying sizes, it was necessary to determine the extraction efficiency of the method across tissue samples of different masses. Pooled biopsy blubber samples were divided into masses of 0.1 g, 0.15 g, 0.2 g, 0.25 g, 0.3 g, 0.35 g, and 0.4 g (all ±0.025 g), each one in either duplicate or triplicate, such that one sample of each mass was unspiked while the other one or two were cold spiked with 100 ng of cortisol. Cortisol was then extracted and measured as discussed above, and the percent recovery calculated for each sample.

There was a significant negative correlation between extraction efficiency and sample mass (linear model, p = 0.006, adjusted R^2^ = 0.46) with efficiencies ranging between 99.88% for the 0.1 g samples down to 63.21% for the 0.4 g samples. The linear model parameters were used to calculate the expected extraction efficiencies for all the biopsy samples based on their mass (see Supplementary Fig. 3 online). These extraction efficiencies were then used to correct the measured cortisol concentrations in each sample to give a final cortisol concentration used for statistical analysis.

### Statistical Analysis

All statistical analyses were carried out using the statistical package, R, version 3.1.2 (R Development Team, 2014). Statistical significance was taken at p = 0.05.

#### Plasma Data

The effect of capture time on the plasma cortisol concentrations (n = 50 where capture time was recorded) was investigated using a Linear Regression model.

#### Blubber Data

The effect of capture time on the blubber cortisol concentrations (n = 50 where capture time was recorded) was also investigated using a Linear Regression model. In addition, Generalised Linear Models (GLMs) with a gamma distribution and a log-link function were used to model the data so as to better take into account the right-skew in the hormone concentrations in the tissue. To investigate annual variability in blubber cortisol concentrations, the annual cycle was divided into four seasons based on the time of sampling: ‘pre-breeding’ (May), ‘breeding’ (June and July), ‘moult’ (August) and ‘other’ (September-March). Biopsy mass, plasma cortisol concentration, sex, season (‘pre-breeding’, ‘breeding’, ‘moult’ and ‘other’), area (Shetland, Orkney, the Moray Firth, the Firth of Forth and the Inner Hebrides) and body condition (girth/length) were used as explanatory variables to model the cortisol concentrations. An interaction term was included between sex and season to take into account the possibility that hormone concentrations may not show the same changing relationship across the seasons in males and females.

Backwards variable selection was performed in order to identify the best subset of variables and interactions to explain the variation in hormone concentrations, and the fit of the final model was assessed (see Supplementary Table S2).

## Additional Information

**How to cite this article**: Kershaw, J. L. and Hall, A. J. Seasonal variation in harbour seal (*Phoca vitulina*) blubber cortisol - A novel indicator of physiological state?. *Sci. Rep.* **6**, 21889; doi: 10.1038/srep21889 (2016).

## Supplementary Material

Supplementary Information
