# Supplementary material for: Seasonal variation in harbour seal (Phoca vitulina) blubber cortisol - A novel indicator of physiological state?
Source: Sci Rep. 2016 Feb 24;6:21889. doi: 10.1038/srep21889 (PMC4764809; doi:10.1038/srep21889)
Supplement: Supplementary Information [file srep21889-s1.pdf]

SUPPLEMENTARY INFORMATION

Seasonal variation in harbour seal *(Phoca vitulina)* blubber cortisol - A novel indicator of physiological state?

Joanna L. Kershaw[1*], Ailsa J. Hall[1]

[1] Sea Mammal Research Unit, Scottish Oceans Institute, University of St Andrews,

St Andrews, Fife. KY16 8LB, UK.

* Corresponding author: jk49@st-andrews.ac.uk

1. **Sample Analysis**

Cortisol extraction from blubber biopsies

Briefly, the blubber biopsies were accurately weighed and homogenized in 1000µl ethanol. Homogenates were then centrifuged at 3,000 *rcf* for 10 minutes and the supernatants collected. These were then evaporated under compressed air while incubating at 25^0C. Two milliliters of ethanol : acetone (4:1) were added to the residue and after vortexing and centrifugation, the solution was again evaporated to leave a new residue. 1ml diethyl ether was added to this residue and after similar evaporation steps, 1ml of acetonitrile was added with 1ml of hexane. The solution was vortexed and centrifuged for 20 minutes. The solvents formed two immiscible layers with hexane on top. The hexane layer was removed, and the acetonitrile layer re-extracted with 1ml hexane, centrifuged for 20 minutes and the final acetonitrile layer aspirated and evaporated. The final residue was centrifuged briefly and then re-dissolved in 500µl phosphate buffered saline (pH 7.5) containing 1% bovine γ globulin [1].

2. **Verifications**

Parallelism Assays

Five plasma samples and six blubber extracts were serially diluted from neat through 1/2, 1/4 and 1/8 with the 0 ng/ml cortisol standard provided in the ELISA kit. In a set of guidelines published for assessing parallelism between bioassay dilution curves derived from ELISAs, logistic-log models were found to describe the data with the greatest accuracy over the widest dilution range [2]. 4 parameter logistic-log models describe, with a high degree of accuracy, standard reference samples and sample curves that display a pronounced sigmoidal shape when plotted on an optical density versus log dilution scale [2]. Here, optical density was modelled against the dilution factor, using a 4 parameter logistic-log model to maintain accuracy across a range of concentrations shown by our data. As the plasma data dilutions ranged across the whole standard curve, between 40 ng/ml to 750 ng/ml, the whole standard curve was used. As the blubber dilution extracts did not range across the whole standard curve, but were concentrated between 5 ng/ml and 75 ng/ml, only the lower end of the standard curve between 0ng/ml and 100ng/ml was used for comparison to assess parallelism with this part of the curve that we are interested in. Statistical comparisons among the standard curve and the extract dilution curves and the plasma dilution curves were carried out

by modelling the dilutions as 4-parameter log-logistic models using the ‘drm’ function in the ‘epicalc’ library in the statistical package R, version 3.1.2 (R Development Team, 2014). Examples for these models are shown in Supplementary Fig. 1. Parameter estimates for each model were then compared to assess if the curves were parallel to each other and to the standard curve. There were no significant differences between the parameter estimates of each plasma dilution curve or the standard curve (p values > 0.1), thus providing strong evidence that these dilution curves and standard curve were parallel to each other. The same statistical comparisons were carried out on the blubber extract dilutions and there were no significant differences between the parameter estimates of each dilution curve or the standard curve (p values > 0.1), with the exception of the upper values ie. the asymptote of the curves. Logistic-log curves can have identical slopes and different asymptotes and do not violate the parallelism rule for accurate quantification of antibody in a sample [2]. Parallelism of these curves supports the assumption that the binding characteristics allow the reliable determination of hormone levels in the diluted blubber extracts and in the plasma samples [3].

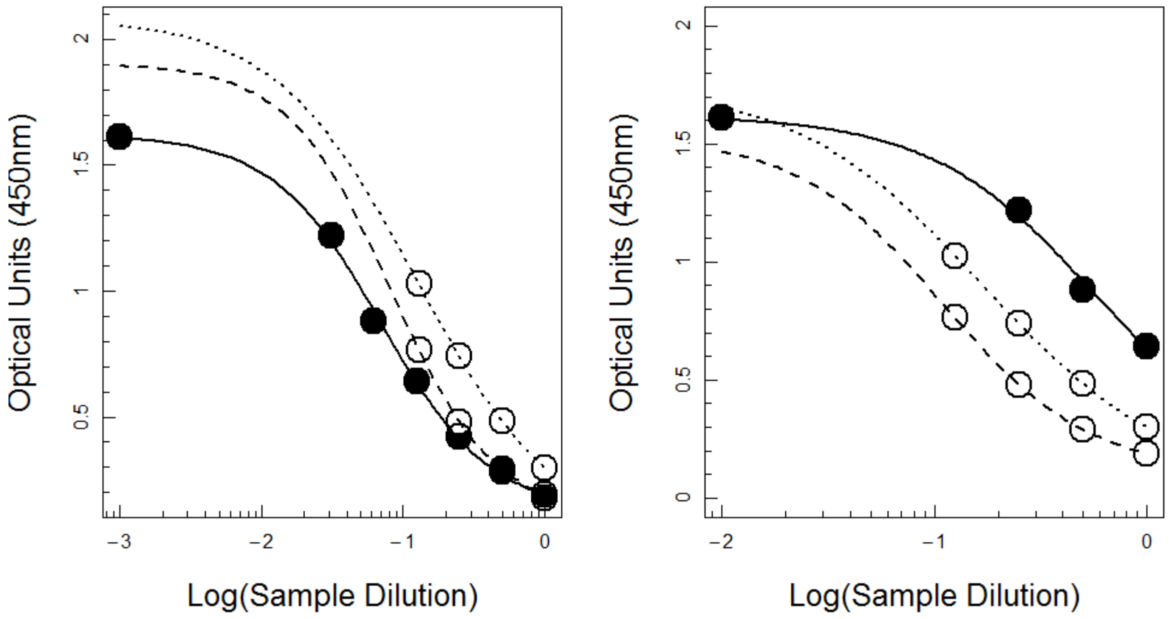

Supplementary Fig. 1. a) Example plot of the cortisol ELISA parallelism validations for two diluted plasma samples. The standard curve is indicated by the closed circles, while the plasma dilutions are indicated by the dashed lines and open circles. b) Example plot of the cortisol ELISA parallelism validations for the diluted blubber sample extracts. The standard curve is indicated by the closed circles, while the two blubber dilutions are indicated by the dashed lines and open circles.

3.2 Matrix Effect Tests

Successful immunologic assays require an optimal pH and ionic strength that promotes specific antibody–antigen complexes while reducing the nonspecific binding of other proteins in the samples that increase assay interference. As the ELISA kit used here was designed for use with serum or plasma samples, the compatibility of the kit with extracts resuspended in PBS with 1% bovine γ globulin was assessed. Equal volumes of each standard were spiked with PBS with 1% bovine γ globulin and assayed in tandem with the unspiked standard curve. The known and the apparent concentrations in the spiked samples should show a positive linear relationship with a slope of approximately 1.0 [4]. The matrix effect test was successful with a slope of 0.99 (Supplementary Fig. 2) (a slope of between 0.8-1.2 was considered acceptable). It was concluded that matrix effects are minimal and this sample diluent is therefore compatible with the immunoassay.

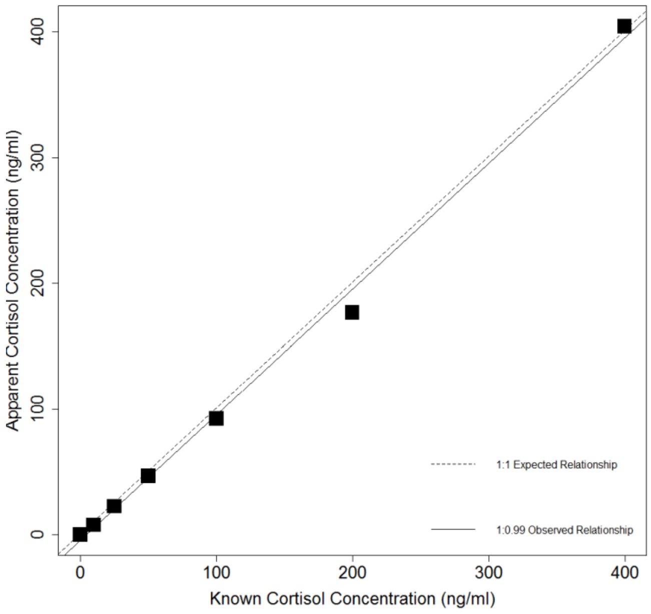

Supplementary Fig. 2. Cortisol ELISA accuracy validations. The linear regression lines for the known against the apparent concentrations are shown for the expected 1:1 relationship and the observed

3.3 Cortisol Recovery from Spiked Samples

As the blubber biopsies were of varying sizes, it was necessary to determine the extraction efficiency of the method across tissue samples of different masses. Pooled biopsy blubber samples were divided into masses of 0.1g, 0.15g, 0.2g, 0.25g, 0.3g, 0.35g, and 0.4g (all ±0.025g), each one in triplicate, such that one sample of each mass was unspiked while the

other two were cold spiked with 100ng of cortisol. Cortisol was then extracted and measured as discussed above, and the percent recovery calculated for each sample.

There was a significant negative correlation between extraction efficiency and sample mass (linear model, $p = 0.006$, Adjusted $R^2 = 0.46$) with efficiencies ranging between 99.88% for the 0.1g samples down to 63.21% for the 0.4g samples (Supplementary Fig. 3). This linear model (Supplementary Fig. 3) was used to calculate the expected extraction efficiencies for all the biopsies samples based on their mass (Equation 1). These extraction efficiencies were then used to correct the measured cortisol concentrations in each sample to give a final cortisol concentration used for statistical analysis.

Equation 1:

Extraction Efficiency = -139.40 x (Biospy Mass) + 118.04

Where -139.40 is the gradient of the relationship and 118.04 is the y intercept.

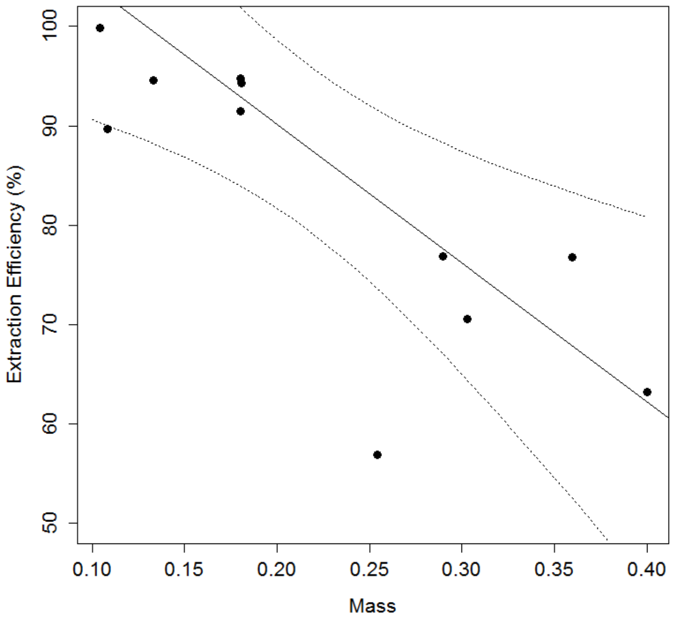

Supplementary Fig. 3. Significant negative relationship between cortisol extraction efficiency and blubber biopsy mass - linear model, $p = 0.006$, Adjusted $R^2 = 0.46$.

3. Statistical Analyses

Blubber Data: First, the largest Generalise Linear Model (GLM) including all the explanatory variables was generated. Then, the dredge function in the MuMIn library in R was used to identify the best subset of variables and interactions to explain the variation in hormone concentrations, and thus to include in the final model. The dredge function is a backwards selection tool that runs models with all possible combinations of the explanatory

variables including both the specified interactions between variables and their respective main effects. The goodness of fit of each model was assessed using the QAIC (quasi-Akaike's information criterion). The QAIC was used instead of the AIC in order to adjust for the over-dispersion in the data. The models were then ranked by their QAIC to determine which combination of variables best explains the relationships in the data. The model with the lowest QAIC value was used for further interpretation as this contained only the variables and interactions of importance. The fit of the final model, and thus its predictive power was evaluated visually by plotting the observed against the fitted values [5]. The final model showed a good relationship between the fitted and the observed values, thus giving confidence in the model fit.

Supplementary Table 1 – Results of the generalised linear model backwards selection process for the 5 models that best explained the variation in the blubber cortisol concentration data.

MODEL	df	QAIC
**Blubber**~Biopsy+Plasma+as.factor(Sex)+as.factor(Season)+as.factor(Location)	11	912.3
**Blubber**~ Biopsy+as.factor(Sex)+as.factor(Season)+as.factor(Location)	10	914.7
**Blubber**~Biopsy+as.factor(Sex)+ as.factor(Location)	8	927.6
**Blubber**~Biopsy+Plasma+as.factor(Sex)+as.factor(Location)	9	927.8
**Blubber**~Biopsy+ as.factor(Season)+as.factor(Location)	9	937.7

Supplementary Table 2 – Mean ± standard deviation of blubber cortisol concentrations by sex across seasons and locations. NB. These means do not consider the combination of factors affecting blubber cortisol as identified by the GLM as being significant in Supplementary Table 1. These data need to be assessed considering the effects of these other factors as well.

	**Female Blubber Cortisol Concentration (ng/g)**			**Male Blubber Cortisol Concentration (ng/g)**		
	**Mean**	**Standard Deviation**	**Sample Size**	**Mean**	**Standard Deviation**	**Sample Size**
**Pre-Breeding**	158.01	± 117.50	13	91.69	± 23.69	10
**Breeding**	149.64	± 34.77	12	120.31	± 35.62	5
**Moult**	1234.72	± 277.70	33	985.25	± 218.21	8
**Other**	203.55	± 101.44	5	115.28	± 86.74	29
**Firth of Forth**	232.89	± 261.95	3	52.35	± 10.31	7
**Inner Hebrides**	162.03	± 67.99	14	170.63	± 97.45	14
**Moray Firth**	104.75	± 0.00	1	88.58	± 18.27	9
**Orkney**	149.64	± 34.77	12	93.51	± 35.18	14
**Shetland**	1234.72	± 277.70	8	985.25	± 218.21	8

References

1. Kellar, N.M., et al., *Determining pregnancy from blubber in three species of delphinids.* Marine Mammal Science, 2006. **22**(1): p. 1-16.
2. Plikaytis, B.D., et al., *Determination of parallelism and nonparallelism in bioassay dilution curves.* Journal of Clinical Microbiology, 1994. **32**(10): p. 2441-2447.
3. Grotjan, H.E. and B.A. Keel, *Date Interpretation and Quality Control*, in *Immunoassay*, E.P. Diamandis and T.K. Christopoulos, Editors. 1996, Academic Press: New York, NY. p. 51-95.
4. Hunt, K.E., R.M. Rolland, and S.D. Kraus, *Detection of steroid and thyroid hormones via immunoassay of North Atlantic right whale (Eubalaena glacialis) respiratory vapor.* Marine Mammal Science, 2014. **30**(2): p. 796-809.
5. Crawley, M.J., *The R Book*. 2007, Chichester, England: John Wiley & Sons, Ltd.
